# Functional Endoscopic Sinus Surgery as a primary modality of treatment for primary and recurrent nasal polyposis

**DOI:** 10.12669/pjms.332.11800

**Published:** 2017

**Authors:** Mohammad Shahid Gohar, Saleem Asif Niazi, Sohail Baber Niazi

**Affiliations:** 1 Dr. Mohammad Shahid Gohar, MBBS, FCPS. Department of ENT, Combined Military Hospital, Multan Cantt, Pakistan; 2 Dr. Saleem Asif Niazi, MBBS, MCPS, FCPS. Department of ENT, Combined Military Hospital, Multan Cantt, Pakistan; 3 Dr. Sohail Baber Niazi, MBBS, FCPS. Department of ENT, Combined Military Hospital, Multan Cantt, Pakistan

**Keywords:** Functional endoscopic sinus surgery, Nasal polyposis, Preferred treatment

## Abstract

**Objectives:**

To describe the efficacy of Functional Endoscopic Sinus Surgery(FESS) in our set up in comparison with other published studies to treat primary and recurrent nasal polyposis.

**Method:**

This descriptive study was conducted in 02 years at Ear Nose Throat Department Combined Military Hospital (CMH) Multan from October 2013 to October 2015. Convenient sample comprising 116 patients of both sexes of age group from 18 to 60 years were selected from ENT Out Patient Department, with documented diagnosis of nasal polyposis that underwent functional endoscopic sinus surgery. Out of 116 patients, 15 (12.9%) had recurrent nasal polyposis while 101 (87.1%) had primary nasal polyposis. Patients were assessed clinically. Preoperative nasal endoscopy and CT scan of nose and paranasal sinuses were performed in every patient to assess the extent of disease and evaluate the surgical anatomy. Patients were followed up 03 monthly, 06 monthly and after 01 year. Clinical signs of nasal polyposis were evaluated by nasal endoscopy at each follow up visit.

**Results:**

There were 116 patients with documented diagnosis of nasal polyposis. Among these, 75 (64.7%) were male and 41 (35.3%) were female patients. Mean age of presentation in males was 39.1 years and that of females was 36.7 years. Only 15 patients (12.9%) developed recurrent disease within a year.

**Conclusion:**

Functional endoscopic sinus surgery is preferred as a primary treatment modality for primary and recurrent nasal polyposis. Mucosal polyps can be carefully debrided, the natural ostia enlarged, the ethmoid sinuses are unroofed, and sphenoid sinuses are opened in nasal cavity and sinus nasal mucosa is mostly preserved due to excellent visualization and surgical technique. Improvement in symptoms with minimal chance of recurrence may be expected in more than 90% patients.

## INTRODUCTION

Nasal polyps are common tear-drop shaped growths that form in nose or paranasal sinuses. These can develop in all paranasal sinuses but the region of middle meatus and osteomeatal complex is most favored. These are often linked to allergies and long term infections especially fungal sinusitis. Most people with nasal polyps have rhinorrhoea, sneezing, anosmia / hyposmia and post-nasal drip. There may be associated deviated nasal septum(DNS), enlarged turbinates or atopies. Generally topical nasal steroid drops with oral antihistamine are highly effective in relieving symptoms. Sometimes short-term systemic steroid courses are also prescribed. However in refractory and uncontrolled cases surgery is the last resort to improve quality of life.

Conventional nasal polypectomy has lost its charm due to high rate of recurrence. Ankema et al.^1^ have found that although 12 weeks of treatment with fluticasone propionate nasal drops reduced the need for sinus surgery in patients with nasal polyposis and chronic rhinosinusitis but 14 out of 27 patients still required surgery. Nowadays FESS has emerged as a treatment of choice for nasal polyposis and chronic rhino sinusitis that is not responsive to aggressive medical treatment. Damm et al.^2^ have demonstrated improvement in quality of life in 85% of patients with a mean follow up time of 31.7 years.

The objective of this study was to assess the efficacy of FESS in the treatment of primary and recurrent nasal polyposis in our setup in comparison with published studies.

## METHODS

A two-year descriptive study was conducted at Combined Military Hospital(CMH) Multan from October 2013 to October 2015, after getting approval from hospital ethical committee and registering the study in our hospital. We selected 116 patients of both sexes of age group of 18 years to 60 years with documented diagnosis of nasal polyposis using convenient sampling technique. Our inclusion criterion for surgery was based upon patients with positive findings of polyposis by CT scan and nasal endoscopy. We excluded those patients who had immunocompromised states due to diabetes mellitus, hypertenion, chronic granulomatous diseases, very young patients and very elderly patients. We performed FESS under general anesthesia in each case after obtaining informed consent. Standard surgical steps were applied in each case according to the extent of disease.

All patients were prescribed nasal steroid drops, oral antihistamines and antibiotics post operatively. Some patients were given oral antifungal medicines also. Every patient was assessed by nasoendoscopy post operatively and regular nasal toilet and debridement of nasal adhesions and crusting was done on each follow up visit. Post operative follow up was carried out at 1^st^ week, 2^nd^ weeks, 1^st^ month, 3^rd^ month, 6^th^ month and one year by all consultants involved in this study. Recurrent disease was defined as a condition of nasal polyposis that occurred in patients who had resolution of disease after surgery but appeared at a later date.

## RESULTS

A total of 116 patients with documented diagnosis of nasal polyposis were included in the study. The group consist 75 (64.7%) male and 41 (35.3%) female patients with an average age range of 18 to 60 years. Mean age of males was 39.1 years and mean age of females was 36.7 years. Almost all patients had nasal obstruction of varying degrees. About 75% patients had anosmia / hyposmia.

CT Scan and nasal endoscopy was carried out in each case, Nasal polyposis was staged in 03 categories i.e. polyps limited to middle meatus, polyps reaching to inferior meatus and polyps reaching to floor of nasal cavity. Seventy two percent were placed in stage-2 and 28% were placed in stage-3. Radiological grading of nasal polyposis by using CT scan was also carried out.

Out of 116 patients who were regularly followed up only 17 (12.0%) patients were diagnosed as cases of recurrence. All patients were examined by using nasal endoscope on every follow up visit. No major post operative complication was noted and minor issues of adhesions and crusting were dealt with at the spot.

## DISCUSSION

Nasal polyps are soft, painless, non cancerous growths on lining of nasal passages and sinuses. They hang down like a bunch of grapes. Polyps respond and shrink using steroid nasal preparations in about 80% of people. Nasal polyps blocking the nose can be removed surgically. But using medication and conventional surgical methods, the polyps recurred in 03 out of 04 patients after an average period of 04 years. Dalzeik et al.^3^ have shown high rate of recurrence of nasal polyposis with simple nasal polypectomy. Messer Kling^4^ introduced mucosal sparing technique using endoscope for sinus surgery. He focused on removing key areas of obstruction to allow normal mucociliary function. Kenedy et al.^5^ used the term FESS to describe the endoscopic technique of simus surgery for treatment of nasal polyposis. Stamberger^6^ justified FESS by arguing that the nose and anterior ethmoids are responsible for almost all infections of frontal and maxillary sinuses. FESS is particularly used at osteomeatal area, clearing diseased air cells and mucosal contact areas. Ventilation and drainage of maxillary and frontal sinuses are re-established via their natural ostia. Steward et al.^7^ showed great improvement in symptoms with severe disease on preoperative CT Scan with FESS. We preserved middle turbinate in all except 05 patients. In those 05 cases we have to remove or cauterize the middle turbinate for better access and visualization. We experienced no major complication during surgery. Suzuki et al.^8^ have demonstrated a very overall low rate of complications of 0.5% after functional endoscopic sinus surgery.

We prescribed post operative nasal steroid preparations, antibiotics and antihistamines to the patients. Oral antifungal medication was prescribed to five patients with fungal sinusitis. Lildholdt et al.^9^ have recommended post operative nasal steroid drops, especially in refractory cases.

The long term follow up reveals that FESS is an operative method of choice for patients with nasal polyposis. Senior et al.^10^ have demonstrated improvement of symptoms in 91.6% cases with FESS with a mean follow up time of 7.8 years. Bolger WE et al.^11^ have reported that FESS is a very beneficial surgical procedure in improving mucociliary transport by decreasing inflammation, oedema and polyp formation.

This study is comparable with the international studies conducted hence it demonstrates that FESS is a treatment modality of choice for both primary and recurrent nasal polyposis. There is superior access and visualization of middle meatus mucosa, uncinate process and infundibulum, which are the sites from which 80% of polyps arise, Bhata Charyya N^12^, Satish N.^13^ report that it is a safe and successful treatment with low morbidity and improved patient comfort.

Both FESS and CT technology has concurrently expanded the indications for sinus surgery according to Emma et al.^14^ There is growing use of new technology of image^15^ guided endoscopic surgery in alleviating all concerns about brain, eyes and major vessels. This type of surgery may be recommended for severe forms of chronic sinusitis, in cases when previous sinus surgery has attended anatomical land marks or when a patient’s sinus anatomy is very unusual.

## CONCLUSION

Functional endoscopic sinus surgery is a preferred modality of treatment for nasal polyposis. It is an efficient and safe modality with minimum morbidity and complication rates. The recurrent rate of nasal polyposis is sufficiently reduced with greater improvement of symptoms and quality of life in more than 90% of cases.
